# Synergistic Pathogenicity by Coinfection and Sequential Infection with NADC30-like PRRSV and PCV2 in Post-Weaned Pigs

**DOI:** 10.3390/v14020193

**Published:** 2022-01-20

**Authors:** Jinyong Zhang, Peng Wang, Changzhan Xie, Zhuo Ha, Ning Shi, He Zhang, Zhuoxin Li, Jicheng Han, Yubiao Xie, Xiangshu Qiu, Yimo Tao, Ningyi Jin, Huijun Lu

**Affiliations:** 1 Key Laboratory of Jilin Province for Zoonosis Prevention and Control, Changchun Veterinary Research Institute, Chinese Academy of Agricultural Sciences, Changchun 130122, China; jinyongnh@163.com (J.Z.); pengwang202112@163.com (P.W.); xiechangzhan2019@163.com (C.X.); hazhuo163@163.com (Z.H.); shiningtcs@126.com (N.S.); hezhangvs@126.com (H.Z.); 13043393036@163.com (Z.L.); 877216599@163.com (Y.X.); qxiangshu@126.com (X.Q.); tym1104597270@163.com (Y.T.); 2 College of Animal Science and Technology, Jilin Agricultural University, Changchun 130118, China; 3 Academician Workstation of Jilin Province, Changchun University of Chinese Medicine, Changchun 130117, China; hjc_0703@163.com; 4 Jiangsu Co-Innovation Center for the Prevention and Control of Important Animal Infectious Disease and Zoonoses, Yangzhou University, Yangzhou 225012, China

**Keywords:** NADC30-like PRRSV, PCV2, coinfection, sequential infection, pathogenicity

## Abstract

Porcine reproductive and respiratory syndrome virus (PRRSV) and porcine circovirus (PCVs) are two major viruses that affect pigs. Coinfections between PRRSV and PCV2 are frequently reported in most outbreaks, with clinical presentations involving dyspnea, fever, reduced feed intake, weight loss, and death in fattening pigs. The NADC30-like PRRSV and PCV2d are the main circulating virus strains found in China. This study determines the impact of NADC30-like PRRSV and PCV2d mono-infection and coinfection on the immune system, organ pathology, and viral shedding in five-week-old post-weaned pigs. Pigs were randomly divided into six groups: PBS, PRRSV, PCV2, PRRSV-PCV2 coinfection (co), and PRRSV-PCV2 or PCV2-PRRSV sequential infections. Fever, dyspnea, decreased feed intake, weight loss, and pig deaths occurred in groups infected with PRRSV, Co-PRRSV-PCV2, and PRRSV-PCV2. The viral load was higher in Co-PRRSV-PCV2, PRRSV-PCV2, and PCV2-PRRSV than those mono-infected with PRRSV or PCV2. Additionally, cytokines (IFN-γ, TNF-α, IL-4, and IL-10) produced by pigs under Co-PRRSV-PCV2 and PRRSV-PCV2 groups were more intense than the other groups. Necropsy findings showed hemorrhage, emphysema, and pulmonary adhesions in the lungs of pigs infected with PRRSV. Smaller alveoli and widened lung interstitium were found in the Co-PRRSV-PCV2 and PRRSV-PCV2 groups. In conclusion, PRRSV and PCV2 coinfection and sequential infection significantly increased viral pathogenicity and cytokine responses, resulting in severe clinical signs, lung pathology, and death.

## 1. Introduction

Porcine reproductive and respiratory syndrome (PRRS) is an infectious disease with a high infectivity rate in pigs. It is caused by the Porcine Reproductive and Respiratory Syndrome virus (PRRSV), an enveloped RNA virus in the genus *Arterivirus* of the family *Arteriviridae* [1]. PRRSV was first reported in the USA in 1987, and then isolated in the Netherlands [2,3]. Genetically, PRRSV is divided into two distinct groups: Betaarterivirus suid 1 (PRRSV-1, known as European genotype) and Betaarterivirus suid 2 (PRRSV-2, known as North American genotype), in which the nucleotide sequence variation of PRRSV-1 and PRRSV-2 is 30–45% [4,5]. In China, PRRSV was first reported in 1996 [6]; and then a new PRRSV characterized by high morbidity and mortality, named highly pathogenic PRRSV (HP-PRRSV), emerged in 2006 [7]. Meanwhile, the NADC30-like PRRSV strain was first detected in 2013. This novel strain spread rapidly and soon became one of the most predominant circulating PRRSV strains in the domestic pig industry, causing substantial economic losses [8,9]. The main clinical symptoms of NADC30-like PRRSV are high fever, cough, anorexia, blue ears, discoloration of the body, increased mortality in piglets, and abortion and stillbirth in sows [10,11]. The NADC30-like PRRSV showed much lower pathogenicity as compared with HP-PRRSV [12].

Porcine circovirus (PCVs) is a non-enveloped DNA virus that contains a single-stranded circular genome belonging to the genus *Circovirus* of the family *Circoviridae* [13,14]. Currently, there are four recognized types of PCVs: porcine circovirus 1 (PCV1), porcine circovirus 2 (PCV2), porcine circovirus 3 (PCV3), and porcine circovirus 4 (PCV4) [14,15,16]. The PCV2 was considered the most pathogenic as it causes porcine circovirus diseases and porcine circovirus-associated diseases (PCVD/PCVAD), with clinical and subclinical presentations in pigs [17,18]. The clinical symptoms of PCVD/PCVAD are porcine dermatitis, nephropathy syndrome (PDNS), post-weaning multisystemic wasting syndrome (PMWS), and porcine respiratory disease complex (PRDC) [14,18,19]. The PCV2 is divided into four subtypes, from PCV2a to PCV2d, of which the PCV2d was prevalent in China [19].

Both PRRSV and PCV2 target the immune system of pigs, impairing pigs’ immune defense against pathogenic microbes and increasing the host’s susceptibility to secondary infections by primary and secondary pathogens [20]. In post-weaned pigs, coinfection rates of PRRSV and PCV2 in lungs with proliferative and necrotizing pneumonia lesions could be as high as 42 and 85.4%, respectively [21,22]. Furthermore, in pigs, coinfection with PRRSV and PCV2 exhibits more severe clinical signs, such as severe dyspnea and lethargy at about 10 days and death at about 20 days after infection. By contrast, mono-infection with PCV2 or PRRSV presents milder clinical symptoms [23]. In addition, coinfection or secondary infection of PRRSV and PCV2 significantly increases the morbidity and mortality of pigs [22]. So far, there is no research on coinfection and secondary infection between NADC30-like and PCV2d. In this study, we analyze the coinfection and sequential infection of NADC30-like and PCV2d in finishing pigs, which may provide a basis for research on the prevention and treatment of these two diseases.

## 2. Materials and Methods

### 2.1. Cells and Viruses

NADC30-like PRRSV and PCV2 (2d) were isolated and preserved by our laboratory. The PK-15 cell lines and Marc-145 cell lines were maintained in the Dulbecco’s modified Eagle’s medium (DMEM; Hyclone, Logan, UT, USA) supplemented with 10% fetal bovine serum (FBS; Hyclone, Logan, UT, USA) and cultured at 37 °C under 5% CO_2_ to propagate PCV2 and PRRSV, respectively.

### 2.2. Animal Inoculation and Samples Collection

A total of 30 five-week-old post-weaned pigs, negative for PRRSV and PCV2 based on nucleic acid and antibody tests, were randomly allocated into six experimental groups (five pigs for each group). The six groups were as follows: a non-infected control group (PBS); PRRSV-infected group (PRRSV); PCV2-infected group (PCV2); PRRSV and PCV2 co-infected group (Co-PRRSV-PCV2); PRRSV and PCV2 sequentially infected group (PRRSV-PCV2); PCV2 and PRRSV sequential infected group (PCV2-PRRSV). Among them, the second infection of the sequential infection group was operated on the 7th day of the first infection. The pigs were challenged with PRRSV (10^3^TCID_50_/mL; intranasal 2 mL+ intramuscular injection 6 mL) or PCV2 (10^4^TCID_50_/mL, intranasal 2 mL+ intramuscular injection 6 mL) or PBS (intranasal 2 mL+ intramuscular injection 6 mL). Blood collection was performed at 0, 3, 7, 10, 14, 21, 28, or 35 (PRRSV-PCV2 group and PCV2-PRRSV group) days post-infection (dpi) for viremia load detection and specific antibody detection. Pigs were euthanized at 28 dpi (PBS group, PCV2 group, PRRSV group, and Co-PRRSV-PCV2 group) or 35 dpi (PRRSV-PCV2 group and PCV2-PRRSV group) for viral load detection, necropsy, and histopathology analysis.

### 2.3. Clinical Symptom Analysis

Clinical signs demonstrating pigs’ infection with PRRSV or PCV2 were recorded. Changes in rectal temperature were recorded for 21 consecutive days. Infected groups’ main clinical symptoms (i.e., mental states, feed intake, emaciation, diarrhea, respiratory symptom, thick coat, and other clinical symptoms) were recorded for 21 days. Average daily gains of the pigs were calculated (Average daily gain [kg/d] = [weight of pigs at the time of death or euthanasia- weight of pigs on day 1 of challenge]/survival time of pigs). The survival rates of each infected group were also recorded and calculated.

### 2.4. Antibody Responses Induced by PRRSV or PCV2

Serum samples were collected for detection of specific antibodies against PRRSV or PCV2 detection at 0, 7, 14, 21, 28, and 35 dpi. Specific antibodies against PRRSV or PCV2 were detected by enzyme-linked immunosorbent assay (ELISA). PRRSV-specific antibodies were detected using an ELISA kit (Ke Qian Biology, Wuhan, China), according to the manufacturer’s protocols. PCV2 specific antibodies were detected using the PCV2-dCap-ELISA Ab kit (Jinnuo Bai tai Biotechnology, Beijing, China), according to the manufacturer’s protocols.

### 2.5. Viremia Detection and Viral Detection in Tissues of PRRSV and PCV2

Blood samples were collected (0, 3, 7, 10, 14, 21, 28, and 35 dpi) for PRRSV and PCV2 virological examination. Pigs from each group underwent euthanasia to examine their macroscopic pathology and for tissue sample collection. Lungs, submaxillary lymph nodes, and inguinal lymph nodes were collected for viral load detection. Total RNA extractions were performed according to the manufacturer’s instructions using RNA TRIzol reagent (Sangon, Shanghai, China). Random primers (primer 9) (Takara, Dalian, China) and M-MLV reverse transcriptase (Promega, Madison, WI, USA) were used for reverse transcription to obtain the cDNA. Using TIANamp Genomic DNA kit (TianGen, Beijing, China), genomic blood samples or tissues were extracted according to the manufacturer’s instructions for PCV2 detection. SYBR Green I quantitative real-time PCR (q-PCR) was used to detect PRRSV and PCV2 viral load. The detection primers were designed using Primer Premier 5 (Table 1).

### 2.6. Cytokine Detection in Serum

Commercial pig enzyme-linked immunosorbent assay (ELISA) kit (Cloud-clone Corp., Wuhan, China) were used to test the levels of interleukins-4 (IL-4), interleukins-10 (IL-10), interferon-γ (IFN-γ), and tumor necrosis factor-α (TNF-α). In addition, cytokine levels were measured according to the manufacturer’s instructions.

### 2.7. Necropsy and Histopathology

Necropsies were performed on 28 dpi (PBS, PCV2, PRRSV, and Co-PRRSV-PCV2 groups) or 35 dpi (PRRSV-PCV2 and PCV2-PRRSV groups). Pathological observations of dissected porcine organs or tissues, such as heart, liver, spleen, lung, kidney, submaxillary lymph nodes, and inguinal lymph nodes, were recorded. In addition, the lungs were fixed with 4% paraformaldehyde and processed for histopathological examination using hematoxylin and eosin (HE) staining.

### 2.8. Statistical Analysis

Statistical analysis and data plotting were performed using GraphPad Prism (version 6.0) software (GraphPad Software Inc., La Jolla, CA, USA). The following *p* values were indicated: * indicates *p* < 0.05, ** indicates *p* < 0.01, and *** indicates *p* < 0.001.

## 3. Results

### 3.1. Clinical Evaluation of Pigs

**Figure 1:**
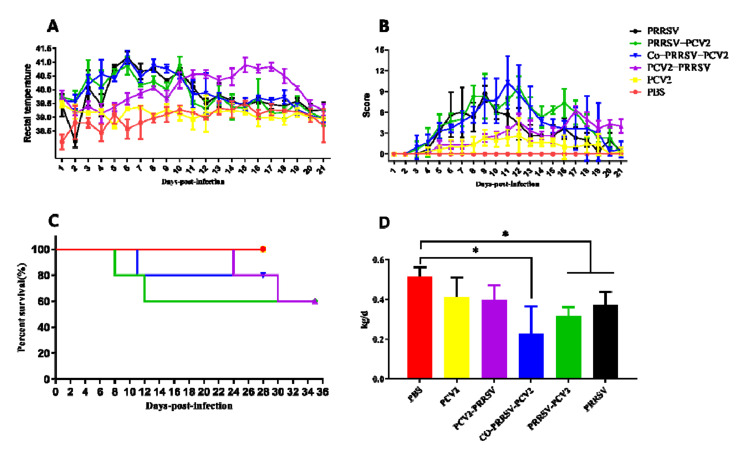
Evaluation of clinical symptoms in pigs after challenged. (**A**) Rectal temperatures of pigs were shown, and the temperature for fever in pigs was set at 39.5 °C; (**B**) clinical symptom scores were recorded for 21 days. Clinical symptoms were assessed, with higher scores indicating more severe clinical symptoms, and 0 representing no obvious clinical symptoms. (**C**) Mortality of pigs after challenge; (**D**) the average daily weight gain of the infected pigs. The following *p* values were indicated: * indicates *p* < 0.05.

Rectal temperature, clinical symptom scores (Table S1), mortality, average daily weight gain, and other clinically relevant data of the challenged pigs were recorded and analyzed carefully. In contrast, rectal temperatures of PRRSV, PRRSV-PCV2, and Co-PRRSV-PCV2 groups increased significantly on the 4th dpi, in which a high rectal temperature lasted for about six days (the temperature for fever in pigs was set at 39.5 °C). The rectal temperature of the PCV2-PRRSV group increased considerably on the third dpi with PRRSV, which lasted for about ten days (Figure 1A). Each group of pigs showed different degrees of clinical signs, including cough, asthma, dyspnea, depression, diarrhea, emaciation, and coarse hair, with observed mortality after infection with PRRSV. According to the results of symptoms scoring, the clinical signs of PRRSV-PCV2, Co-PRRSV-PCV2, and PCV2-PRRSV infected groups were more severe than the other groups (Figure 1B). The mortality rate of the PRRSV-PCV2, PCV2-PRRSV, and Co-PRRSV-PCV2 groups was 40, 40, and 20%, respectively (Figure 1C). The average daily gain of pigs in each infected group was lower than that in the PBS group. In comparison, the average daily gain of pigs in the Co-PRRSV-PCV2, PRRSV-PCV2, and PRRSV groups was significantly lower than that in PBS infected group (Figure 1D).

### 3.2. PRRSV and PCV2 Antibody Response of the Infected Groups

**Figure 2:**
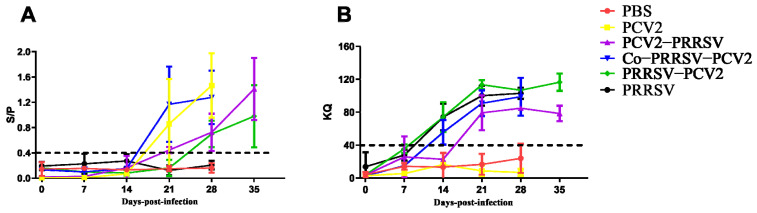
Specific antibody of PCV2 and PRRSV were monitored. (**A**) Trends level of PCV2-specific antibody, S/P < 0.4 were considered negative, and S/P ≥ 0.4 were considered positive; (**B**) the trend of PRRSV-specific antibodies changes with the time of PRRSV infection in pigs, KQ < 40 were judged to be negative, and KQ ≥ 40 were judged to be positive.

Serum samples were collected at 0, 7, 14, 21, 28, and 35 dpi to monitor specific antibodies for PCV2 or PRRSV. Specific antibodies for PCV2 were detected at 21 dpi with a rapid increase in Co-PRRSV-PCV2 and PCV2 groups, reaching the highest level at 28 dpi. By contrast, the PCV2-specific antibodies of the PCV2-PRRSV infected group showed a slower increase, although its antibody levels were achieved at almost the same rate as PCV2 and Co-PRRSV-PCV2 groups. Meanwhile, PCV2-specific antibodies produced in the PRRSV-PCV2 group were lower than that in PCV2 group and Co-PRRSV-PCV2 group at 28 dpi (Figure 2A). PRRSV-specific antibodies were detected at 14 dpi, and the highest level of PRRSV-specific antibodies appeared on the 21st day and remained until the end of the experiment. The PRRSV-specific antibody values were lower in the PCV2-PRRSV infected group than in the other PRRSV infected groups (Figure 2B).

### 3.3. Viremia Detection and Viral Detection in Tissues of PRRSV and PCV2

**Figure 3:**
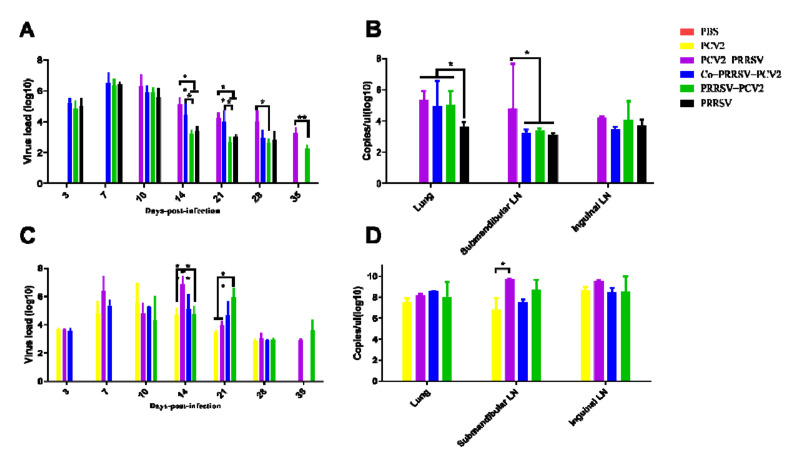
Viremia detection and viral detection in tissues of PRRSV and PCV2. (**A**) Trends in viremia with PRRSV, with peak viral loads 3–7 days post challenge; (**B**) the viral load of PRRSV in lung and lymph node was measured; (**C**) time-course of viremia with PCV2, high viral load at 7–14 dpi; (**D**) the viral load of PCV2 in lung and lymph node. The following *p* values were indicated: * indicates *p* < 0.05, ** indicates *p* < 0.01.

The qPCR results on the viremia of PRRSV showed that viral load was highest on 7 dpi in the Co-PRRSV-PCV2, PRRSV-PCV2, and PRRSV group. However, the viral load decreased rapidly to 14 dpi and was lowest at 28 dpi. The viral loads of the Co-PRRSV-PCV2 group were significantly higher than those in the PRRSV-PCV2 and PRRSV-infected groups on 14 dpi and 21 dpi. The viral load of the PCV2-PRRSV group was highest at 3 dpi infection with PRRSV, which then rapidly declined but was significantly higher than that in other PRRSV-infected groups on 14, 21, 28, and 35 dpi (Figure 3A). Examination of PRRSV viral loads in the lungs revealed that the viral loads of Co-PRRSV-PCV2, PRRSV-PCV2, and PCV2-PRRSV groups were significantly higher than those of the PRRSV infected group. Moreover, the viral load of PRRSV in the inguinal lymph nodes of the PCV2-PRRSV group showed substantially higher levels than the other PRRSV-infected groups (Figure 3B).

Viremia of PCV2 in each infection group was detected, and the viral load was found to be highest on 7–14dpi of infection in groups PCV2, PCV2-PRRSV, Co-PRRSV-PCV2. The PRRSV-PCV2 group had the highest viral load on 14 dpi and then rapidly declined (Figure 3C). In addition, viral loads of PCV2 in the lungs, inguinal lymph nodes, and submandibular lymph nodes were detected, and the results showed that the viral load of the PCV2-PRRSV group in the inguinal lymph nodes was significantly higher than that of the PCV2 group, with no significant differences among the others (Figure 3D).

### 3.4. Cytokine Concentrations in Serum

**Figure 4:**
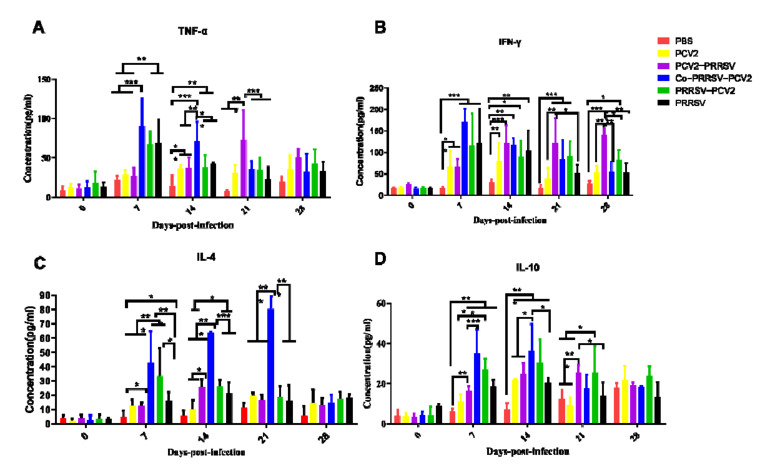
Cytokine detection in serum of each experimentally infected group. (**A**) TNF-α detection in serum, the cytokine of TNF-α was significantly TNF-α cytokine levels were significantly higher in pigs infected with PRRSV; (**B**) IFN-γ detection in serum, cytokines were significantly elevated 7–14 days after infection with PRRSV, and high levels of cytokines persisted for 21 days after PCV2-PRRSV infection with PRRSV; (**C**) IL-4 detection in serum, cytokine levels were significantly higher in the Co-PRRSV-PCV2 group than in the other groups on 7–21 dpi; (**D**) IL-10 detection in serum, cytokines were significantly elevated 7–14 days after infection with PRRSV. The following *p* values were indicated: * indicates *p* < 0.05, ** indicates *p* < 0.01, and *** indicates *p* < 0.001.

Cytokine detection using ELISA showed an increase in cytokines at different levels after PRRSV infection. TNF-α cytokine concentrations were significantly higher in the Co-PRRSV-PCV2, PRRSV-PCV2, and PRRSV groups at 7 dpi. However, the Co-PRRSV-PCV2 and PCV2-PRRSV groups showed significantly higher levels among other groups at 14 and 21 dpi, respectively (Figure 4A). The cytokine concentration of IFN-γ increased rapidly after PRRSV infection. The cytokine concentrations of Co-PRRSV-PCV2, PRRSV-PCV2, and PCV2-PRRSV groups were significantly higher than that of the control group (Figure 4B). Detection of IL-4 cytokines in the serum showed that the Co-PRRSV-PCV2 infection group had significantly higher concentrations of cytokines than the other groups on 7, 14, and 21dpi (Figure 4C). The results of IL-10 cytokine assays revealed that the cytokine concentrations in each infection group showed various degrees of elevation, and the concentrations of Co-PRRSV-PCV2 and PRRSV-PCV2 groups were higher than in the other infection groups at 7 and 14 dpi (Figure 4D).

### 3.5. Anatomy and Histopathology

**Figure 5:**
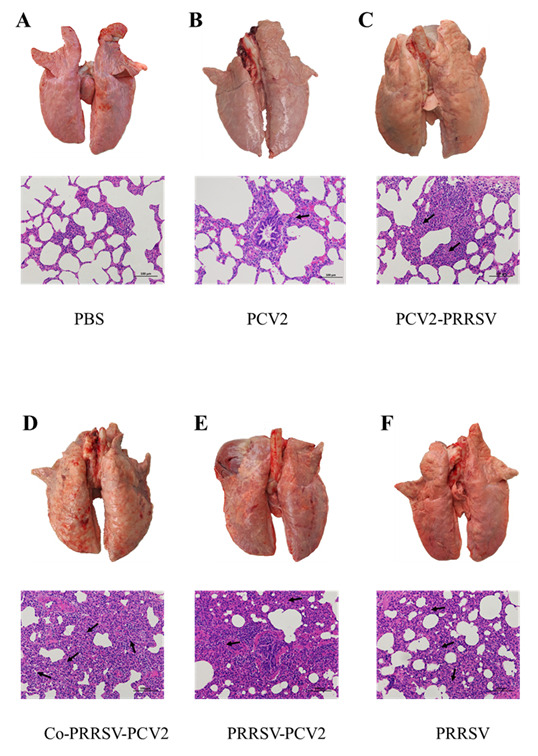
Representative histopathological sections of lung from each infected group (200×). (**A**) Healthy control group (PBS group); (**B**) PCV2 infected group, low-grade interstitial pneumonia (black arrow); (**C**) PCV2-PRRSV infected group, the alveoli were smaller and the lung interstitium was thickened (black arrow); (**D**) Co-PRRSV-PCV2 infected group, hemorrhagic spots were present in the lung, severe interstitial pneumonia (black arrow) with effacement of alveoli; (**E**) PRRSV-PCV2 infected group, hemorrhagic spots, severe interstitial pneumonia (black arrow) with effacement of alveoli were present in the lung; (**F**) PRRSV infected group, interstitial pneumonia (black arrow), partial alveolar effacement.

We found that lungs from the PCV2-infected group only showed a slight enlargement of the interstitium, with no other noticeable gross lesions (Figure 5B). On the other hand, pigs coinfected or sequentially infected with PRRSV showed severe lesions in lung tissues. In particular, Co-PRRSV-PCV2 and PRRSV-PCV2 groups showed severe hemorrhages, emphysema, and sarcoid changes, with some pigs developing pulmonary adhesions, among others. Pathological sections showed widening of the lung interstitium and partial alveolar effacement (Figure 5D,E), in addition to some pathological changes such as hemorrhages and edema in the lymph nodes. The symptoms in the PCV2-PRRSV and PRRSV groups were relatively mild compared to those in the Co-PRRSV-PCV2 and PRRSV-PCV2 groups, with emphysema in some pigs and presence of sarcoid changes reflecting histological sections of the lungs, showing interstitial lung enlargement and partial alveolar effacement (Figure 5C,F). Pathological examinations showed that Co-PRRSV-PCV2 and PRRSV-PCV2 groups had the most severe lesions, followed by the PRRSV, PCV2-PRRSV, and PCV2 groups.

## 4. Discussion

PCV2 and PRRSV mainly attack the immune system of pigs, resulting in immune deficiency, and then lead to respiratory symptoms, fever, and other clinical symptoms in pigs [20]. Since 2013, several outbreaks of PRRSV in vaccinated pigs have been reported in Central China, such as in the Henan province, where sows infected with PRRSV developed reproductive failure, and weaned piglets developed clinical symptoms such as dyspnea. Previous works on the nucleic acid of this PRRSV revealed a high similarity to the NADC30 strains in the United States, designated as the NADC30-like PRRSV [8,24]. Compared with HP-PRRSV, piglets infected with NADC30-like PRRSV showed much lower pathogenicity, with clinical signs such as dyspnea, anorexia, and fever [9]. Meanwhile, the PCV2 causes the Post-weaning Multisystemic Wasting Syndrome (PMWS), characterized by systemic, respiratory, enteric, and reproductive lesions. It is also the primary agent of the Porcine Dermatitis and Nephropathy Syndrome (PDNS). Clinical indications from both syndromes can be replicated to varying degrees [14,25]. Clinically, NADC30-like PRRSV and PCV2 can be detected simultaneously [26], but the co-pathogenicity of NADC30-like PRRSV and PCV2 to pigs is still unclear. This study intends to use NADC30-like PRRSV and PCV2d (both epidemic strains in China) to study the impact of coinfection and sequential infection of the two viruses on infection parameters in pigs.

Previous studies have shown that highly pathogenic (HP)-PRRSV and PCV2 coinfection and secondary infection can increase the clinical symptoms of pigs, and especially that the HP-PRRSV and PCV2 sequential infection could exhibit synergistic aggravating clinical effects [6]. Eclercy and colleagues found that Modified-live virus (MLV)-like-PRRSV coinfection with PCV2 can boost clinical signs in specific-pathogen-free (SPF) pigs [27]. Commonly, PRRSV and PCV2 coinfection in pigs predispose the animal to polymicrobial infections, thereby aggravating clinical signs and morbidity [21,28,29]. Pigs co-inoculated with PRRSV and PCV2 exhibited severe dyspnea, lethargy, occasional icterus, and even death, while PCV2-infected pigs developed lethargy, sporadic icterus, and exudative epidermitis. Meanwhile, PRRSV-inoculated pigs only showed dyspnea and mild lethargy [23]. Our clinical data showed that clinical symptoms of Co-PRRSV-PCV2, PRRSV-PCV2, and PCV2-PRRSV groups are more severe when compared to a single infection with either PRRSV and PCV2. Pigs co-infected and sequentially infected with NADC30-like PRRSV and PCV2 showed persistent high fever, tachypnea, dyspnea, sensory depression, anorexia, weight loss, and even death. Therefore, PRRSV and PCV2 coinfection and secondary infection can aggravate the clinic symptoms compared with the PRRSV and PCV2 infection groups. 

We also monitored the specific antibodies against PRRSV or PCV2 after HP-PRRSV and PCV2 inoculation in experimental pigs. Our results revealed that antibody titers increased over time; for PCV2 specific antibodies, the mono-infection showed higher levels than those of the coinfected or secondarily infected groups; for PRRSV specific antibodies, the PRRSV group, PRRSV-PCV2 group, and Co-PRRSV-PCV2 group were generally concordant, and higher than in the PCV2-PRRSV group. While viral loads in the blood decreased over time in all groups, the viral loads in the coinfection or secondary infection remained higher than single infections. Previous studies have shown that PRRSV can prolong the duration of PCV2 viremia and shedding time in vivo. The PRRSV can also affect the infection kinetics of PCV2a and PCV2b during coinfection [28].

Furthermore, MLV-like PRRSV coinfection with PCV2 in SPF pigs showed that the virulence of PRRSV and PCV2 increased significantly [27]. In our study, PCV2-specific antibodies of Co-PRRSV-PCV2 and PCV2 infected groups grew rapidly and higher than other infected PCV2 groups. We found that sequential infection of PRRSV and PCV2 delayed the production of PCV2-specific antibodies. PRRSV-specific antibodies showed generally consistent increasing trends in all groups of pigs infected with PRRSV, but only the PCV2-PRRSV group was lower than other groups. 

An inflammatory storm can emerge when animals infected with virus, IL-4, IL-10, TNF-α, and IFN-γ serve as some representative cytokines of the adaptive immune response when swine are infected with PRRSV or PCV2 [6,29,30,31]. High concentrations of TNF-α can cause severe pathological damage that can significantly cause death in laboratory animals following the viral challenge [6,31,32]. In our study, the concentration of TNF-α in Co-PRRSV-PCV2, PRRSV-PCV2, and PCV2-PRRSV groups significantly increased after PRRSV infection in pigs. This may explain the death of pigs in the Co-PRRSV-PCV2, PRRSV-PCV2, and PCV2-PRRSV groups. IFN-γ can inhibit the replication of PRRSV and PCV2 [32]. In our study, the concentration of IFN-γ in all groups was increased after infection with the virus. However, the concentrations of IFN-γ in Co-PRRSV-PCV2 and PCV2-PRRSV were significantly higher than in other groups. We also found that the viral load of PCV2 with PRRSV in these two groups was higher than that in the other groups, explaining the increase in IFN-γ concentration in each infection group. The IL-4 and IL-10 were important cytokines in adaptive immunity, indicating the degree of the immune system after viral inoculation. Our results showed that the concentrations of IL-4 and IL-10 in Co-PRRSV-PCV2 and PRRSV-PCV2 groups were significantly higher than the other groups, suggesting that adaptive immune responses were robust in these infection groups. The upregulated expression of inflammatory factors may cause an inflammatory storm, which may affect the survival rate and clinical symptoms of pigs.

Pigs co-inoculated with PRRSV and PCV2 showed more severe proliferative interstitial pneumonia and hepatic lesions. In contrast, PRRSV-inoculated pigs only had moderate proliferative interstitial pneumonia without bronchiolar or hepatic lesions or lymphoid depletion [23]. Histopathological analyses in pigs inoculated with HP-PRRSV and/or PCV2 revealed necrosis and/or lymphoid depletion in the lymph nodes, lymphocytic infiltration of the liver portal areas, thromboses in the small pulmonary blood vessels and alveolar capillaries, and varying degrees of interstitial pneumonia. Such histological lesions were most severe in the HP-PRRSV/PCV2 group [6]. A previous study also showed that SPF pigs coinfected with MLV/MLV-like PRRSV and PCV2 had extensive thickening of the alveolar septa and increased heterogeneity in alveolar size due to focal atelectasis and alveolar emphysema [27]. Previous reports showed that pathogenicity can improve when the NADC30-like PRRSV isolate is coinfected with PCVs [26]. Our results found that severe lesions occurred in the anatomical organs of the Co-PRRSV-PCV2 and PRRSV-PCV2 groups, especially in the lungs. These pathological lesions include hemorrhages, emphysema, and sarcoid changes. Histopathological examination of the lungs revealed alveolar effacement, alveolar wall thickening, and lymphocyte infiltration.

## 5. Conclusions

In conclusion, pigs coinfected or secondarily infected with PRRSV and PCV2 showed more severe clinical symptoms than the single-infected group. Pigs infected with PRRSV can enhance the pathogenic of PCV2, in which severe organ damage manifests as pulmonary hemorrhage and sarcoidosis in the lungs. Additionally, pigs infected with PCV2 will likely show an increase in the pathogenic of PRRSV. Pigs coinfected or sequentially infected with PRRSV and PCV2 produced a relatively strong cytokine response.

## Figures and Tables

**Table 1:** Primers detection of PRRSV or PCV2 by q-PCR.

Name of Primers	Primers (5′→3′)	Size of Products (bp)
PRRSV-qPCR-F	GAAGAAGAATAAGAATAGAAACCCG	195
PRRSV-qPCR-R	GGCAAACTAAACTCCACAGTGTAAC	
PCV2-qPCR-F	AAAAGCAAATGGGCTGCTAA	83
PCV2-qPCR-R	TGGTAACCACCCACCACTT	

## Data Availability

All available data are presented in the article.

## Supplementary Materials

The following are available online at https://www.mdpi.com/article/10.3390/v14020193/s1, Table S1: Reference form for clinical score, Table S2: Clinical symptom score data for pigs in each group.
