# Supplementary material for: Synergistic Pathogenicity by Coinfection and Sequential Infection with NADC30-like PRRSV and PCV2 in Post-Weaned Pigs
Source: Viruses. 2022 Jan 20;14(2):193. doi: 10.3390/v14020193 (PMC8877551; doi:10.3390/v14020193)
Supplement: Supplementary file 1 [file viruses-14-00193-s001.zip › viruses-1527595-supplementary.pdf]

Supplementary material files

Table S1 Reference form for clinical score

	Pig No.	Mental states	Feed intake	Vomit	Diarrhea	Respiratory symptom	Erythra	Purple ears	Emaciation	States of thick coat	Other symptoms
Group											

Note: The content of each item is 0-3 points, with 0 representing no clinical symptoms, 1 representing mild clinical symptoms, 2 representing moderate clinical symptoms, and 3 representing severe clinical symptoms. Each pig needs to be carefully observed and scored daily. Taking pig No. 1 as an example, it was eventually necessary to sum the individual scores to give a total score of pig No. 1, and then the figure was made along with the scores of the other pigs within the same group.

Table S2 Clinical symptom score data for pigs in each group

dpi	Groups																	
	PBS			PCV2			PCV2-PRRSV			Co-PRRSV-PCV2			PRRSV-PCV2			PRRSV		
1	0	0	0	0	0	0	0	0	0	0	0	0	0	0	0	0	0	0
2	0	0	0	0	0	0	0	0	0	0	0	0	0	0	0	0	0	0
3	0	0	0	0	0	0	0	0	0	0	0	3	2	0	0	0	0	0
4	0	0	0	1	0	0	0	0	0	1	1	3	4	1	0	1	1	0
5	0	0	0	1	0	1	1	2	1	4	2	4	6	3	3	4	4	2
6	0	0	0	1	0	2	1	2	1	4	3	4	4	4	6	7	8	2
7	0	0	0	1	0	2	1	2	1	5	3	6	5	4	6	7	9	2
8	0	0	0	2	0	2	2	0	2	5	7	5	10	7	8	6	7	3
9	0	0	0	3	3	1	2	2	3	4	9	10	12	6	7	10	8	8
10	0	0	0	2	1	3	3	3	2	9	4	10	6	7	6	7	6	5
11	0	0	0	3	3	1	4	3	3	12	6	13	8	8	7	7	7	3
12	0	0	0	0	3	5	4	4	6	12	4	10	8	11	10	7	3	3
13	0	0	0	1	2	2	2	5	3	4	6	10	5	8	7	4	1	3
14	0	0	0	2	1	2	3	3	2	5	4	5	4	5	7	3	2	3
15	0	0	0	2	1	2	2	4	2	5	3	4	6	7	6	3	4	1
16	0	0	0	3	0	0	4	4	4	3	3	5	9	5	8	4	5	2

17	0	0	0	1	1	1	6	7	6	3	1	7	5	5	8	2	1	4
18	0	0	0	2	2	0	6	4	5	2	0	9	2	5	5	3	2	1
19	0	0	0	3	1	0	3	5	3	1	0	8	2	2	3	0	0	1
20	0	0	0	0	0	0	4	4	5	0	0	1	1	2	4	2	1	3
21	0	0	0	0	0	1	5	4	3	2	0	0	0	0	0	0	1	0

Note: The score in the table is the sum of all clinical symptom scores of a pig.
